# Spatiotemporal Associations and Molecular Evolution of Highly Pathogenic Avian Influenza A H7N9 Virus in China from 2017 to 2021

**DOI:** 10.3390/v13122524

**Published:** 2021-12-15

**Authors:** Dongchang He, Min Gu, Xiyue Wang, Xiaoquan Wang, Gairu Li, Yayao Yan, Jinyuan Gu, Tiansong Zhan, Huiguang Wu, Xiaoli Hao, Guoqing Wang, Jiao Hu, Shunlin Hu, Xiaowen Liu, Shuo Su, Chan Ding, Xiufan Liu

**Affiliations:** 1 Animal Infectious Disease Laboratory, College of Veterinary Medicine, Yangzhou University, Yangzhou 225009, China; virolog@outlook.com (D.H.); gumin@yzu.edu.cn (M.G.); bioyusy@outlook.com (X.W.); wxq@yzu.edu.cn (X.W.); bioyyy@outlook.com (Y.Y.); gu1220jinyuan@163.com (J.G.); biocreater@outlook.com (T.Z.); hgwu80@163.com (H.W.); xlhao@yzu.edu.cn (X.H.); 1986guoqing@163.com (G.W.); hujiaohot@163.com (J.H.); slhu@yzu.edu.cn (S.H.); xwliu@yzu.edu.cn (X.L.); 2 Jiangsu Co-Innovation Center for Prevention and Control of Important Animal Infectious Diseases and Zoonosis, Yangzhou University, Yangzhou 225009, China; shoveldeen@shvri.ac.cn; 3 Jiangsu Key Laboratory of Zoonosis, Yangzhou University, Yangzhou 225009, China; 4 College of Veterinary Medicine, Nanjing Agricultural University, Nanjing 210095, China; ligru2018@163.com; 5 Department of Avian Diseases, Shanghai Veterinary Research Institute, Chinese Academy of Agricultural Sciences, Shanghai 200241, China

**Keywords:** avian influenza virus, H7N9, hemagglutinin, evolution, highly pathogenic, epidemiology, phylogeography

## Abstract

Highly pathogenic (HP) H7N9 avian influenza virus (AIV) emerged in China in 2016. HP H7N9 AIV caused at least 33 human infections and has been circulating in poultry farms continuously since wave 5. The genetic divergence, geographic patterns, and hemagglutinin adaptive and parallel molecular evolution of HP H7N9 AIV in China since 2017 are still unclear. Here, 10 new strains of HP H7N9 AIVs from October 2019 to April 2021 were sequenced. We found that HP H7N9 was primarily circulating in Northern China, particularly in the provinces surrounding the Bohai Sea (Liaoning, Hebei, and Shandong) since wave 6. Of note, HP H7N9 AIV phylogenies exhibit a geographical structure compatible with high levels of local transmission after unidirectional rapid geographical expansion towards the north of China in 2017. In addition, we showed that two major subclades were continually expanding with the viral population size undergoing a sharp increase after 2018 with an obvious seasonal tendency. Notably, the hemagglutinin gene showed signs of parallel evolution and positive selection. Our research sheds light on the current epidemiology, evolution, and diversity of HP H7N9 AIV that can help prevent and control the spreading of HP H7N9 AIV.

## 1. Introduction

H7N9 avian influenza virus (AIV) first emerged in the Yangtze River Delta (YRD) region of China in 2013. It is a novel zoonosis that has resulted in at least five human waves of epidemics and 1568 cases, including 616 deaths (http://www.fao.org/ag/againfo/programmes/en/empres/H7N9/situation_update.html, last accessed on 14 December 2021). H7N9 was low pathogenic (LP) in its early stage, infecting poultry asymptomatically. However, in the summer of 2016, it acquired a four amino acid insertion in the cleavage site of the hemagglutinin (HA) protein, resulting in a shift to high pathogenicity (HP). HP H7N9 infection resulted in about 50% mortality in infected humans [1,2]. Additionally, HP H7N9 caused an almost 100% lethality in infected chickens, resulting in severe losses to the poultry industry [3,4,5].

The H7N9 virus was first detected in live bird markets (LBM) but was rarely found in poultry farms [3]. However, it later spread to poultry farms from LBM through unknown ways. Poultry trade between LBMs, rather than wild bird migration, may have played a critical role in the dissemination of H7N9 [6]. HP H7N9 spread rapidly from the south to the north in chicken flocks, and also possibly by live poultry trade transmission during wave 5 [6,7,8]. To mitigate the impact of HP H7N9 to the poultry industry, a nationwide vaccination campaign with H5/H7(Re-1) bivalent inactivated vaccine was launched in September 2017 [9,10,11]. To address antigenic drift and better match the antigenicity of the prevalent strain, the H7N9 Re-2 vaccine replaced the Re-1 vaccine in December 2018 [10]. In July 2020, the Re-3 vaccine replaced the Re-2 vaccine. Currently, the prevalence of LP H7N9 AIV is uncommon, whereas HP H7N9 AIV is still prevalent. Although the prevalence of the HP H7N9 virus in poultry has been significantly reduced and human infection also has been largely eliminated [10,12,13], the H7N9 virus has not been eradicated from poultry [14,15]. Since the vaccines had been deployed to control HP H7N9, vaccine-evading variants invariably developed under vaccination pressure, resulting in the accelerated evolution of the HP H7N9 [16,17,18]. The persistent circulation of HP H7N9 is still a serious threat to public health and the poultry industry. Given the possibility of cross-species transmission and the development of rapid mutations in response to vaccination, understanding its prevalence is a public health priority.

Active surveillance of HP H7N9 AIV is essential to gain a better understanding of its evolutionary diversity and spatial diffusion. Since it emerged in China, H7N9 has been classified into two major lineages: the Yangtze River Delta (YRD) lineage and the Pearl River Delta (PRD) lineage [19]. HP H7N9 belonged to the YRD lineage and was initially enzootic in the south of China. Subsequently, it quickly spread westward to adjacent provinces (Guangxi and Yunnan) and then to Northern China during the wave 5 outbreak [20]. Nonetheless, recent studies reported HP H7N9 AIVs in Central (Anhui [16]) and Southern (Yunnan and Guangdong [17]) China between 2017 and 2019, suggesting that HP H7N9 AIVs may not be only limited to Northern China after wave 6.

Influenza viruses undergo continual genomic changes resulting in antigenic drift and shift, particularly in the HA gene, leading to vaccination failure by antigenic mismatch and even altering the characteristic of receptor-binding [21,22,23]. Adaptive mutations are fundamental for the generation and spreading of beneficial substitutions that increase viral fitness in particular parallel molecular evolution under natural selection [24]. Previous studies demonstrated that A125T and M/L256I were significantly involved in parallel changes with the LP-to-HP evolution of H7Nx AIV [25]. In another study, R130K, V177A, L217Q, and M227I on HA1 protein were under parallel evolution in the human isolated LP H7N9 HA protein [26]. These sites around the RBS and antigenic sites evolved in parallel, implying that they may affect both antigenicity and receptor binding specificity. Currently, HP H7N9 adaptation and parallel evolution require investigation.

Considering that the risk of HP H7N9 AIV to public health is continuous, understanding the evolution and phylodynamics of HP H7N9, as well as the genetic characteristic is important. Therefore, we combined epidemiological studies with phylogenetic analysis to investigate the geographic and temporal distribution, genetic evolution, population dynamics, and amino acid sites under positive selection and parallel mutations of HP H7N9 AIV in China. Our findings shed light on the *status quo* genetic diversity and evolution of the HP H7N9 virus. 

## 2. Materials and Methods

### 2.1. Sample Collection and Virus Isolation

Routine surveillance against the live birds market for the AIV was conducted from October 2019 to April 2021. The collected specimens from poultry were stored in 1 mL phosphate-buffered saline mixed with four antibiotics (1000 units/mL penicillin, 1 mg/mL streptomycin, 0.5 mg/mL kanamycin, and 0.25 mg/mL gentamicin). Additionally, swabs or tissues were collected from ill or dead poultry at clinics or farms. All of the specimens were transported to the laboratory within 24 h and frozen at −80 °C. The supernatant (0.2 mL/egg) from squeezed samples or tissue homogenate specimens after centrifugation was inoculated into 10-day-old specific-pathogen-free (SPF) embryonated chicken eggs age 48–96 h at 35 °C. Allantoic fluids were harvested and determined by hemagglutination tests following the procedures according to the standards manual of the World Organization for Animal Health (https://www.oie.int/fileadmin/Home/eng/Health_standards/tahm/3.03.04_AI.pdf, last accessed on 14 December 2021). All experiments involving live H7N9 virus were conducted in negative-pressure isolators equipped with HEPA filters in animal biosecurity level-3 facility following the institutional bio-safety manual (CNAS BL0015).

### 2.2. RNA Extraction and Genome Sequencing

Viral RNA extraction from H7-positive allantoic fluids was performed with an Easy-Pure Viral DNA/RNA Kit (TransGen, Beijing, China) following the manufacturer’s instructions. Viral RNA was reverse transcribed with a 12-bp primer (5′-AGCAAAAGCAGG-3′). Polymerase chain reactions (PCR) were performed using universal primers described by Hoffmann et al. [27]. PCR products were purified with the TaKaRa Agarose Gel DNA Purification Kit (TaKaRa, Dalian, China). The purified PCR products were sequenced using an ABI 3730 automatic sequencer (Tsingke, Nanjing, China).

### 2.3. Data Collection and Down-Sample

Sequence data were collected from public databases (NCBI and GISAID) and cleaned as described in a previous study [18]. Sequences collected from different sources were pooled together and aligned using MAFFT (v7.453) [28]. Then, BioAider [29] was used to remove highly similar sequences of the LP H7N9 AIV HA gene with a 99.5% threshold. For HP H7N9 AIV, duplicate sequences obtained with the same date and location were removed as well. HP H7N9 AIVs were obtained from the six waves (wave 4 to 9) since its emergence, including wave 4 (*n* = 2, October 2015 to September 2016), wave 5 (*n* = 169, October 2016 to September 2017), wave 6 (*n* = 35, October 2017 to September 2018), wave 7 (*n* = 56, October 2018 to September 2019), wave 8 (*n* = 8, October 2019 to September 2020), and wave 9 (*n* = 8, October 2020 to April 2021). 

### 2.4. Phylogenetic Analysis

RAxML-NG (v1.0.1) was used to construct maximum likelihood (ML) trees [30]. The general time reversible (GTR) model with four gamma categories, was selected as the best-fit substitution model according to the Bayesian information criterion (BIC) value with ModelFinder [31] in the PhyloSuite [32]. The reliability of the phylogenetic tree was estimated with 1000 bootstraps. The ML phylogenetic tree was inspected by linear regression using TreeTime (v0.8.1) [33] to identify sequences with incorrect sampling dates or abnormal root-to-tip distance, which were subsequently removed from the alignments. 

### 2.5. Molecular Evolutionary Analysis

The time-resolved tree was inferred using the Markov chain Monte Carlo (MCMC) framework applied in Bayesian Evolutionary Analysis Sampling Trees (BEAST, v1.10.4) [34]. The GTR+G4 nucleotide substitution model was chosen as the best fit model based on the result of Modelfinder. An uncorrelated relaxed molecular clock model and Bayesian SkyGrid coalescent tree prior were chosen [35]. The lengths of the MCMC chain were set to 200 million generations and trees were collected every 20,000 steps. Tracer (v1.7.1) was used to inspect the convergence (effective sample size > 200) of the log file. TreeAnnotator analyzed the maximum clade credibility (MCC) tree with common ancestor heights following the burn-in of the first 10% trees. The SkyGrid reconstruction plot was implemented to investigate the historical population dynamics of H7N9. The ggtree [36] and ggplot2 [37] packages in R (v4.0.5) were used to visualize trees.

### 2.6. Geographic Trait Association Test (BaTS)

To quantify geographic clustering in HP H7N9 transmission, we obtained the posterior distribution of HA trees sampled by the BEAST program after a first 10% burn-in. Then, the phylogenetic trait association test was performed in the Bayesian tip association significance testing (BaTS v0.9) software [38]. China was divided into four geographical divisions: South (Jiangsu, Shanghai, Anhui, Hubei, Hunan, Zhejiang, Fujian, Taiwan, Jiangxi, Guangdong, Guangxi, Hongkong, Macau, and Hainan), North (Heilongjiang, Jilin, Liaoning, Inner Mongolia, Beijing, Tianjin, Hebei, Shanxi, Henan, and Shandong), Northwest (Shaanxi, Gansu, Qinghai, Ningxia, Xinjiang), and Southwest (Chongqing, Sichuan, Guizhou, Yunnan, Tibet). The geographic trait of each sequence was assigned based on its sampling division (province). The geographical clustering of tips in the HA phylogenies was assessed statistically using three phylogeny-trait association tests (association index, AI; parsimony score, PS; and maximum clade, MC) in BaTS. A significance level of less than 0.05 was accepted.

### 2.7. Adaptation and Parallel Evolution

We evaluated amino acids under molecular adaptation and parallel evolution in HP H7N9 AIV using Hyphy (Hypothesis Testing using Phylogenies, v2.5.2), Treesub programs (https://github.com/tamuri/treesub, last accessed on 14 December 2021) and ProtParCon [39]. To determine which amino acids in the HA coding region were under positive selection, we used Hyphy to estimate the ratio of non-synonymous to synonymous substitution rates (dN/dS) [40,41]. Since the Mixed Effects Model of Evolution (MEME) [42] considers that selection pressure for each site is pervasive or episodic along the entire phylogenetic tree, we employed MEME to detect diversifying selection in HP H7N9 viruses. Significant levels of *p* < 0.05 were considered as an indicator of positive selection in MEME. Treesub was used to infer amino acid substitutions along the branches and ProtParCon was used to evaluate the sites that underwent parallel and convergent evolution among the internal node by reconstructing ancestral nodes to the tips based on sequences. PyMol (v2.5) was used to map the amino acid substitutions against the 3D crystal structure of the HA protein (Protein Data Bank code: 6D7U) [43].

## 3. Results

### 3.1. HP H7N9 AIV Isolation and Sequencing 

**Figure 1:**
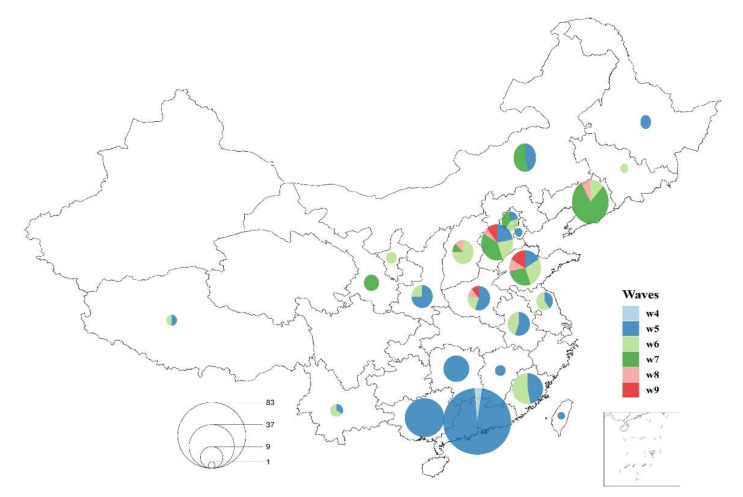
Spatial and temporal distribution of collected high pathogenicity (HP) H7N9 AIV samples since 2016. The pie chart shows the composition of the outbreak waves (periods) and the circle size represents the number of isolated strains.

From October 2019 to April 2021, we collected 10 strains of H7N9 AIVs from diagnosed poultry birds in Northern China (Hebei, Shandong, Shanxi, and Henan) (Table 1 and Figure 1). Two of the 10 strains were obtained from October 2019 to September 2020 and the remaining eight strains were obtained from October 2019 to April 2021. All the collected viruses belong to the YRD lineage and share the same molecular motif (KRTARG) at their cleavage sites, which is a highly pathogenic characteristic of avian species. All the viruses were isolated from chicken, including nine strains isolated from layer chickens and one strain from a broiler chicken. Notably, three of the 10 strains were recovered from sick birds injected with the H5 + H7 bivalent inactive vaccine. We did not detect any viruses from the waterfowl. According to the isolation information of the published sequences, similarly to LP H7N9, HP H7N9 also generally exhibited seasonality (Figure S1), being more prevalent in spring and winter than in summer. Additionally, more HP H7N9 strains were isolated than LP during the summer (Figure S1).

### 3.2. Phylogenetic and Population Dynamic Analyses 

**Figure 2:**
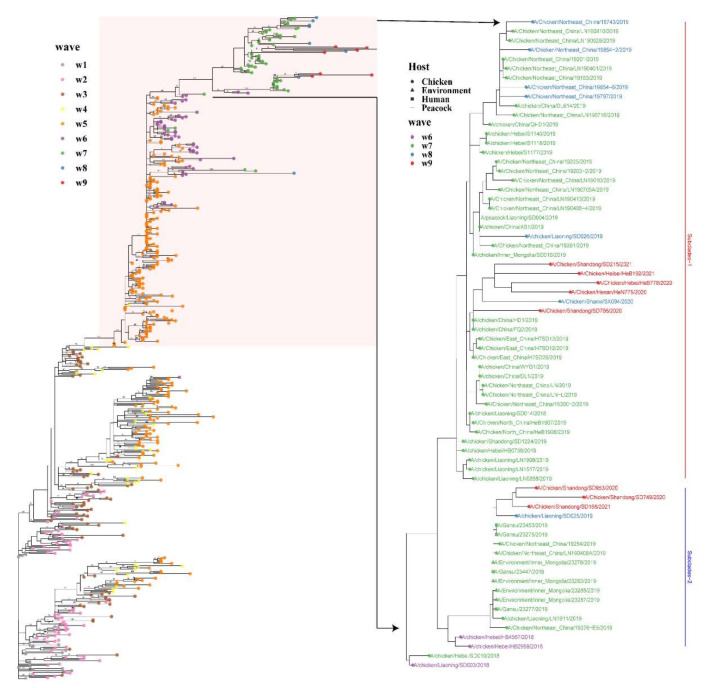
Maximum likelihood (ML) tree of H7N9 AIV. The area highlighted in pink indicates the HP branch. The nodes are plotted based on the bootstrap values. The tips of the phylogenetic tree are colored based on the outbreak waves (periods). The tree on the right panel contains the viruses isolated since 2018.

**Figure 3:**
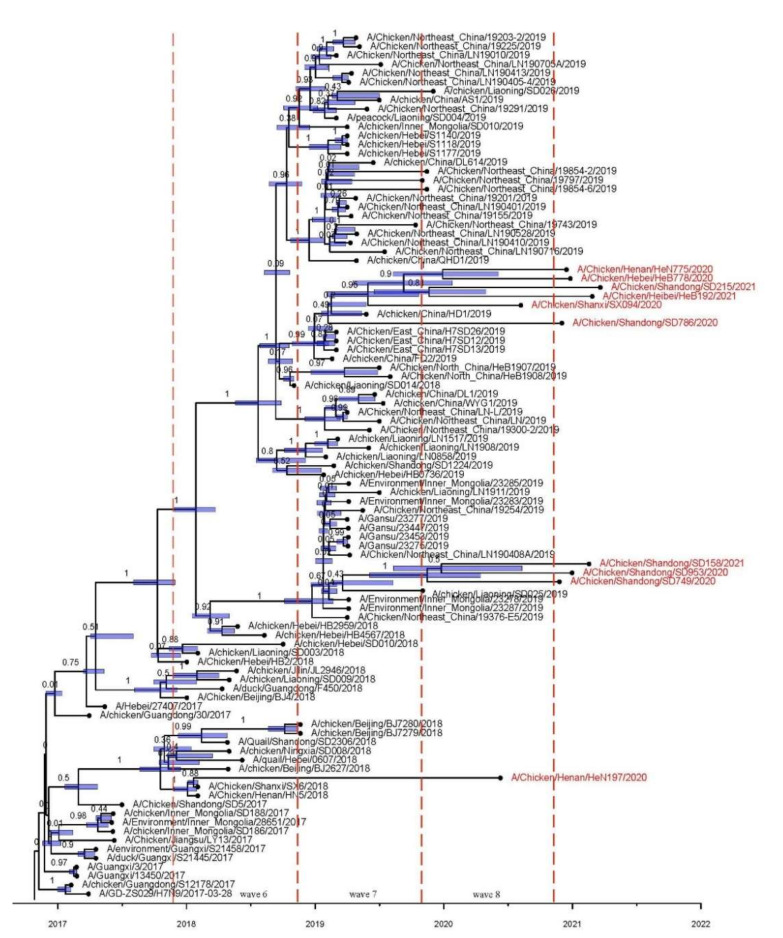
Time-resolved maximum clade credibility (MCC) tree of HA genes. Posterior values are displayed on the tree branches. The internal node highlighted in blue indicates 95% HPD of tree height. The tips of the phylogenetic tree colored in red indicate viruses isolated in our laboratory from October 2019 to April 2021.

**Figure 4:**
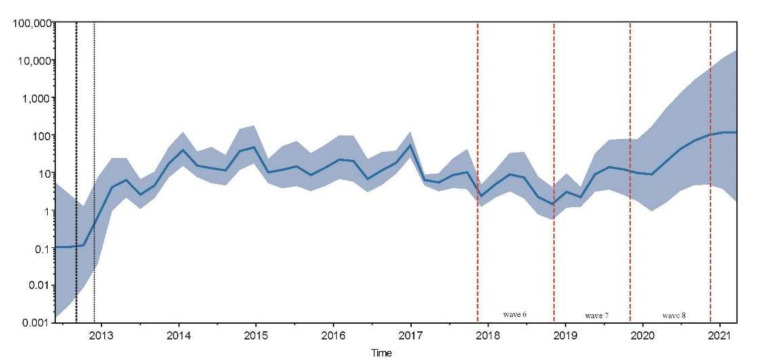
The population size of H7N9. The solid blue curve stands for the median value of the viral population. The blue colored area corresponds to the credibility interval based on 95% highest posterior density (HPD).

The ML phylogenetic tree was used to plot root-to-tip divergence against the sampling date and revealed that the H7N9 AIV sampling date was sufficient to correct the molecular clock in the subsequent time-resolved analysis (Figure S2, R = 0.94). The ML tree and MCC tree showed that all the 278 HP H7N9 AIV viruses in China belonged to the YRD lineage. In general, the HP H7N9 AIV branch exhibited both ladder- (in wave 5) and comb-like (post wave 5) topology (Figure 2). Two major subclades (subclade-1 and subclade-2), which have been formed since January 2018, were continuously increasing and accounted for the majority of isolated strains after wave 7. All three H7N9 virus strains isolated from vaccinated birds after October 2020 were clustered within these two major subclades (Figure 3). The time to the most recent common ancestor (tMRCA) of these two subclades was estimated to be 2018.027 (1 October 2018). Nonetheless, a few strains isolated in waves 7 and 8 clustered with other branches. According to the demographic history of H7N9 circulating in China, the median of population size fluctuated during the nine waves (Figure 4). Since the H7N9 Re-1 vaccine was introduced in poultry herds in September 2017, the viral population size generally decreased and reached a plateau (mean: 1.977, 95% HPD: 5.657–0.761) at the end of wave 6. However, the population size sharply increased onwards of wave 7 and might reach the peak around 2020 (mean: 26.641, 95% HPD: 104.261–7.106). 

### 3.3. Spatial and Temporal Dynamics of HP H7N9 AIV

A total of 574 HA sequences were used in the following analysis, including 296 LP and 278 HP viruses. The spatial and temporal distribution of HP H7N9 AIV showed that the HP H7N9 virus was first detected in the Guangdong province in wave 4 and caused an epidemic (83/278 HP strains isolated in Guangdong) in Pearl River Delta in wave 5 (Figure 1), then rapidly spread to Central and Northern China. Since then, HP H7N9 AIV has been persistently detected in Northern China, while being less prevalent in Southern China. In wave 6, we found that the HP H7N9 virus was discovered for the first time in Jilin province. Since then, the provinces surrounding the Bohai Sea in the North, including Liaoning, Hebei, and Shandong, were the main prevalent areas. Additionally, the HP H7N9 AIV also has been persistently reported in Central (Henan, two cases in wave 6 and one case in wave 8) and Northwest China (Shanxi, one case in wave 6 and one case in wave 8; Shaanxi, one case in wave 6; Gansu, four cases in wave 7) since wave 5. Intriguingly, HP H7N9 AIV was reported less in Southern and Eastern China since wave 6. The phylogenies suggested that HP viral sequences originating from the same location tend to cluster. We used the parsimony score (PS) and association index (AI) statistics in the BaTS program to test for the presence of geographical structure. The places of isolation were used as traits and represented by four locations: Northern China, Southern China, Northwest China, and Southwest China. We found that HP H7N9 has considerable phylogeographic clustering in Northern (*p* = 0.009) and Southern China (*p* = 0.009) (Table S2). Though HP H7N9 migrated to the north from the south, resulting in wave 5, it has been circulating mostly in Northern China while less in the east and south of China after wave 5, implying a strong geographical structure and current localized evolution.

### 3.4. Natural Selection and Parallel Evolution

**Figure 5:**
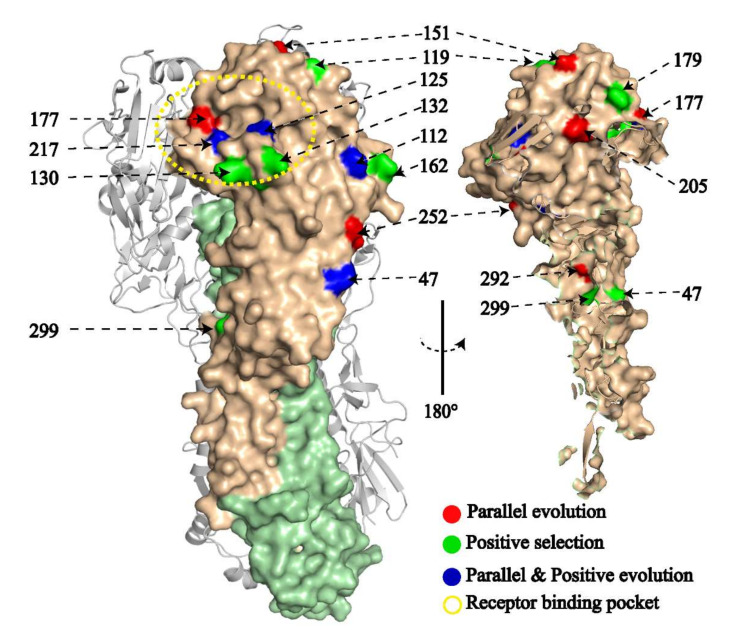
H7N9 HA protein three-dimensional structural map. HA1 is shown in light orange, while HA2 is shown in light green (H7 numbering, PDB: 6D7U).

We evaluated parallel evolution by examining the amino acid substitutions along the HA phylogenetic tree of HP H7N9 AIVs isolated since 2017 (Figure S3 and Table S3). A total of 14 amino acids evolved in parallel, including nine sites on the HA1 protein (R47K, P112S, A151T, V177I, G196E, V205I, Q217L, R252K, and A292T) and five on the HA2 protein (T323I, K389E, D441N, E445K, and V501I). Additionally, V/T125A was detected as part of convergent evolution. MEME found 19 HA sites under positive selection for HP H7N9 AIVs, including 12 on HA1 (sites 47, 61, 112, 119, 125, 130, 132, 162, 179, 196, 217, and 299) and seven on HA2 (sites 323, 324, 478, 507, 510, 511, and 543). Importantly, sites 47, 112, 125, 196, 217, and 323 are under positive selection and parallel evolution. Among these identified substitutions, sites 125, 130, and 132 were discovered simultaneously in the 130-loop of the RBS pocket and antigenic site A (Figure 5). 

## 4. Discussion

Previous studies demonstrated that the domestic poultry trade plays an important role in the transmission of AIV in China [6,20,44]. Particularly, the spreading of highly pathogenic HP H7N9 posed a severe threat to public health and resulted in large economic losses in poultry farms. However, few studies on HP H7N9 dissemination and prevalence in China have been conducted since 2017. In this study, we isolated 10 strains of HP H7N9 AIV from October 2020 to April 2021. The geographical distribution of positive samples revealed that H7N9 might mainly circulate in Northern and Central China, especially in the provinces (Liaoning, Hebei, and Shandong) surrounding the Bohai Sea since wave 6. Population dynamic analysis revealed that the median of population size increased sharply during 2019 with a seasonal tendency in each wave. 

Despite previous studies finding that the spread of H7N9 among domestic chicken is geographically continuous and likely linked to the intensity of live poultry movement [6,44,45], our study revealed that HP H7N9 AIVs most likely followed a single unidirectional invasion followed by local circulation in the north of China rather than reinvasion via poultry trade. In addition, our analysis also reveals important epidemiological features of HP AIV H7N9 in China. The geographical association observed in the phylogenies suggests that HP AIV H7N9 might be transmitted at high levels within local poultry populations and that once it is introduced in an area, it tends to establish local lineages that evolve in situ. Another reason for the reduced impact of long-distance movement and spread of HP H7N9 AIVs (phylogenies exhibit a geographical structure compatible with high levels of local transmission) could be restrictions on long-distance live poultry shipments caused by the COVID-19 pandemic. Since the COVID-19 spread in China at the start of 2020, the consequences of movement control interfering with the circulation and spreading of HP H7N9 are largely unknown. 

In terms of the host, H7N9 was initially detected in live bird markers predominantly in chicken populations. Since then, H7N9 AIVs mainly circulated in domestic poultry and humans rather than in wild birds. Only a small proportion of cases were detected in domestic ducks, pigeons, quails, and other wild birds in the first five epidemic waves [46,47,48,49]. Recent studies showed that the HP H7N9 virus was still detected in ducks and peacocks after wave 5 [16,17]. We found HP H7N9 AIVs primarily in chicken, especially from layers and partially from broilers, indicating that the HP H7N9 mainly infects the longer-lived layers or breeders in the farming industry. 

Many viral evolutionary behaviors, such as cross-species transmission, drug resistance, and host immune evasion, exhibit parallel adaptation. As a result, at least some aspects of virus evolution and emergence are repeatable and predictable [24]. Additionally, since the HA protein is the primary target of neutralizing antibodies [50] and determines specificity for efficient virus transmission between individuals and between species [51], parallel/convergent evolution on HA could not only alter the receptor binding affinity but also affect antigenicity. In this study, we found several parallel/convergent mutations on HA1, including four in the RBS (125 in 130-loop, 151 in 150-loop, 177 in 190-helix, and 217 in 220-loop). Among them, 125 evolved in a convergent (V/T125A) and divergent (V125A/T) manner. Regarding receptor specificity, the HP H7N9 viruses mostly retained dual receptor binding [52,53] features despite the Q217L mutation [3,9,54], which is capable of switching the specificity to the human-type receptor in H2 [55], H3 [56], H4 [57], and H5 influenza virus. It was previously shown that mutations V125T, S134P, A151T, and L217Q in H7N9 occurred around the HA receptor pocket since 2019 increasing the affinity for avian-type sialic acids while decreasing the affinity for human-type sialic acids [18]. Yin et al. observed a similar result using a solid-phase direct binding assay [16]. Besides Q217L, the G177V attributes the dual-receptor properties of the LPAI H7N9 virus [58]. Of note, we report here for the first time, V177I under parallel evolution that emerged in some human sources of HP strains in waves 5 and 6, indicating that the HP H7N9 virus may be able to repeatedly acquire this adaptive mutation in humans. V177I has been reported accountable for the alteration of the dual-receptor-binding tropism [54]. Additionally, our research also identified several novel mutations (S127N/R, R130K/M, S132T/L, G133E/R, S134P, T179I, and A210V) around the receptor-binding pocket. These identified parallel and convergent replacements might play important roles during the multiple adaptations of HP H7N9 in poultry under vaccination, which are intriguing and deserving of further investigation. 

We also detected several amino acid sites under parallel/convergent and positive evolution at HA antigenic sites. Among them, 112, 125, 130, and 132 were in antigenic Site A; 119, 151, 177, and 179 were in antigenic Site B; 299 was in antigenic Site C; 162 and 205 were in antigenic Site D; 47 was in antigenic Site E. Notably, since wave 6, V125T (also under positive selection) and A151T have been fixed in the receptor-binding site (RBS) and antigenic site, resulting in two novel N-glycosylation sites at 123 (NGTT, 130-loop, antigenic Site A) and 149 (NATF, edge of 150-loop, antigenic Site B)(Figure S4). It has been shown that A125T and A151T mutations not only completely abolished human-like receptor binding [59] but also facilitated the H7N9 virus escaping from vaccine-induced immunity [16]. Besides, sites 125/130 mutations induced a 5-fold difference in the neutralizing activity against the field virus [60]. Of note, the antigenicity of HP H7N9 AIV in recent years has drifted rapidly and significantly under intensive vaccination [10,12,16,17,18]. Therefore, the antigenicity of the circulating HP H7N9 virus needs to be closely monitored, and the antigenic composition of vaccines needs to be updated as needed. The effect on the antigenicity of HP H7N9 virus by these parallel/convergent and positive substitutions is still unclear. 

Overall, the retrospective epidemiology and evolutionary analysis summarized the rapid evolution, spatial and temporal distribution of HP H7N9 AIV in China. Surveillance studies in poultry and wild birds are needed to monitor the geographical spread and seasonal patterns of H7N9 HP AIV infections as well as the detection of novel mutations of the viruses. The increased N-glycosylation sites accompanied with the related parallel/convergent mutation sites provided a clue for the evolution of the ongoing prevalence of HP H7N9 AIV.

In summary, our study found that the HP H7N9 AIV phylogenies have a strong geographical structure, with the virus circulating mainly in Northern China and tending to form local lineages. With the size of the viral population growing, two major subclades were constantly expanding. We also discovered that several amino acid sites on the hemagglutinin gene have undergone parallel evolution and positive selection. 

## Figures and Tables

**Table 1:** HP H7N9 AIVs isolated from October 2019 to April 2021.

Full Name	Cleavage Site	Wave	Province	Location	Vaccine	Avian Species	Source	Date
A/Chicken/Henan/HeN197/2020	KRTARG	w8	Henan	Farm	unknown	Layer	Diagnosis	9 June 2020
A/Chicken/Shanxi/SX094/2020	KRTARG	w8	Shanxi	Farm	unknown	Layer	Diagnosis	6 August 2020
A/Chicken/Shandong/SD749/2020	KRTARG	w9	Shandong	Farm	unknown	Layer	Diagnosis	24 November 2020
A/Chicken/Shandong/SD786/2020	KRTARG	w9	Shandong	Farm	H5+ H7	Layer	Diagnosis	1 December 2020
A/Chicken/Henan/HeN775/2020	KRTARG	w9	Henan	Farm	unknown	Broiler	Diagnosis	14 December 2020
A/Chicken/Hebei/HeB778/2020	KRTARG	w9	Hebei	Farm	unknown	Layer	Diagnosis	25 December 2020
A/Chicken/Shandong/SD953/2020	KRTARG	w9	Shandong	Farm	unknown	Layer	Diagnosis	30 December 2020
A/Chicken/Shandong/SD158/2021	KRTARG	w9	Shandong	Farm	H5 + H7	Layer	Diagnosis	16 February 2021
A/Chicken/Hebei/HeB192/2021	KRTARG	w9	Hebei	Farm	H5 + H7	Layer	Diagnosis	25 February 2021
A/Chicken/Shandong/SD215/2021	KRTARG	w9	Shandong	Farm	unknown	Layer	Diagnosis	20 March 2021

## Data Availability

The viral sequences for this study can be found in the Genbank database (accession numbers are available from Supplementary Material Table S1).

## Supplementary Materials

The following are available online at https://www.mdpi.com/article/10.3390/v13122524/s1, Table S1: Viral accession numbers, Table S2: The results of BaTs, Table S3: HA1 adaptation analysis, Figure S1: Seasonality of HP and LP H7N9 AIVs, Figure S2: Analysis of root-to-tip divergence, Figure S3: Amino acid substitutions detected by Treesub, Figure S4: The number of HA N-glycosylation sites against outbreak waves.
