# Supplementary figures and images for: Spatiotemporal Associations and Molecular Evolution of Highly Pathogenic Avian Influenza A H7N9 Virus in China from 2017 to 2021
Source: Viruses. 2021 Dec 15;13(12):2524. doi: 10.3390/v13122524 (PMC8705967; doi:10.3390/v13122524)

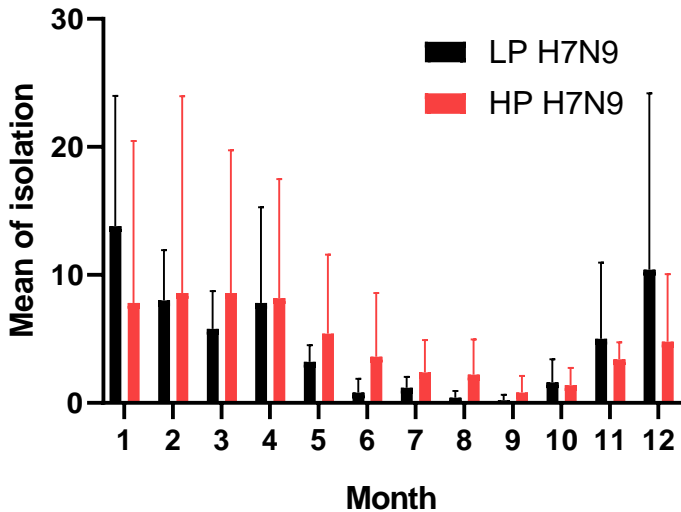

Supplement: Supplementary file 1 [file viruses-13-02524-s001.zip › Figure S1. Seasonality of HP and LP H7N9 AIV.pdf]

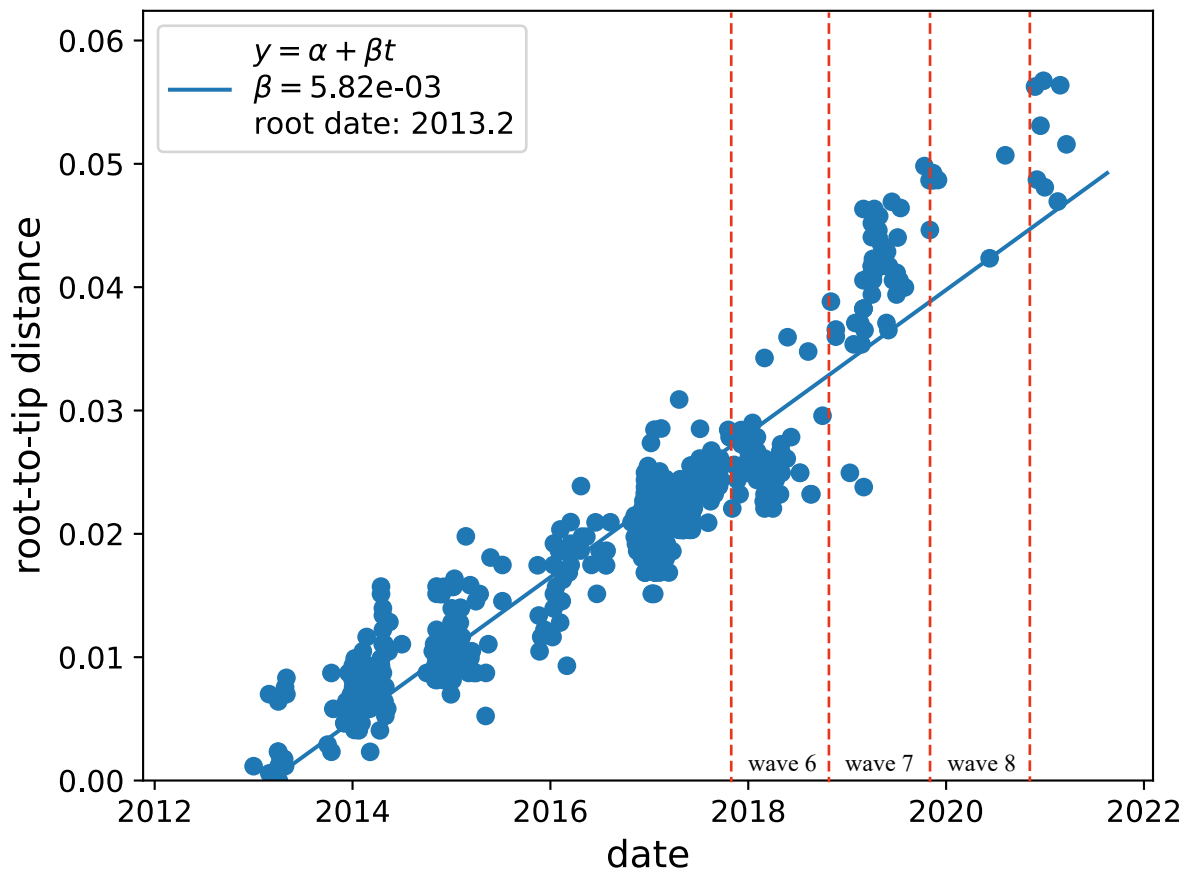

Supplement: Supplementary file 1 [file viruses-13-02524-s001.zip › Figure S2. Analysis of root-to-tip divergence.pdf]

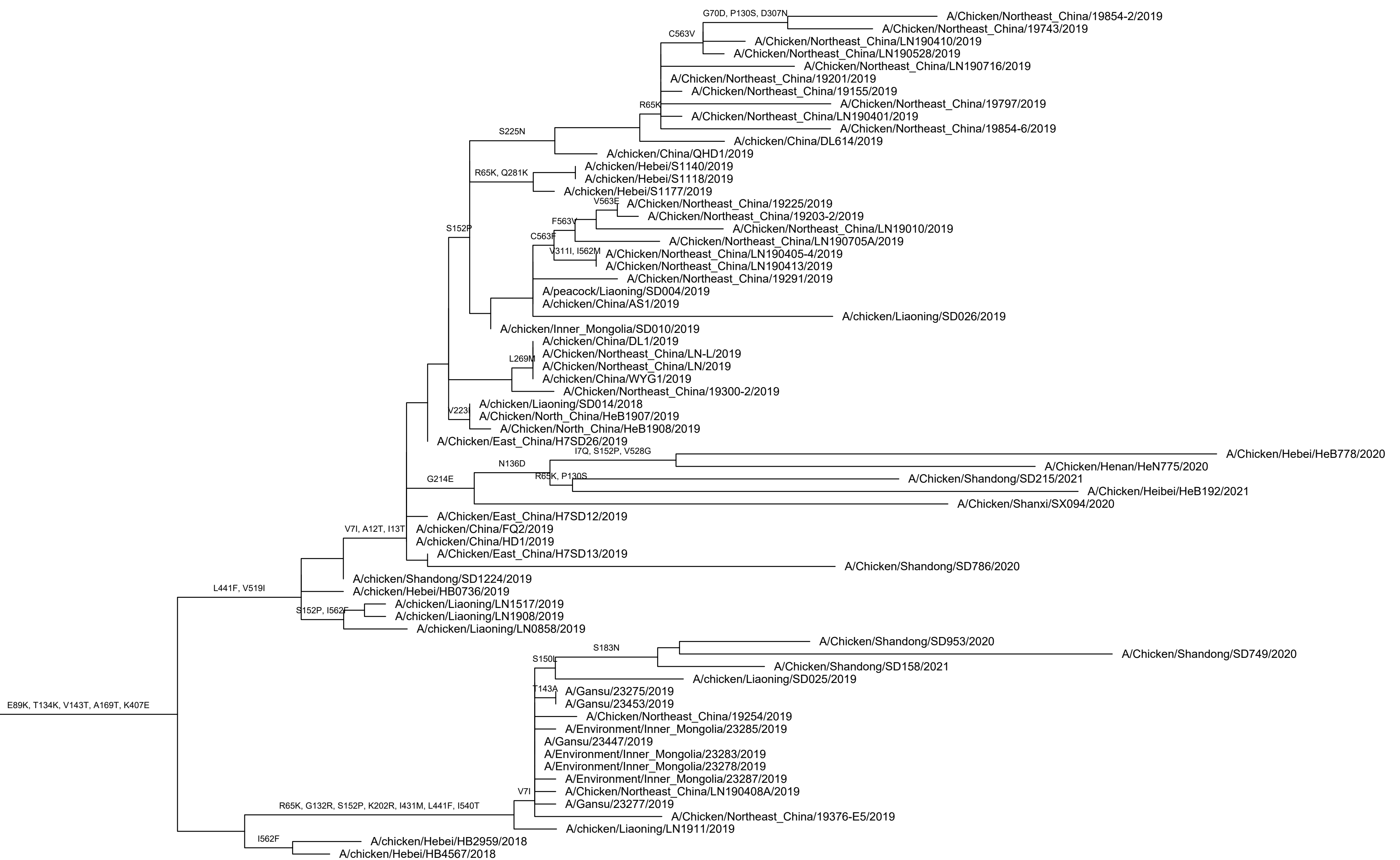

Supplement: Supplementary file 1 [file viruses-13-02524-s001.zip › Figure S3. Amino acid substitutions detected by Treesub.pdf]

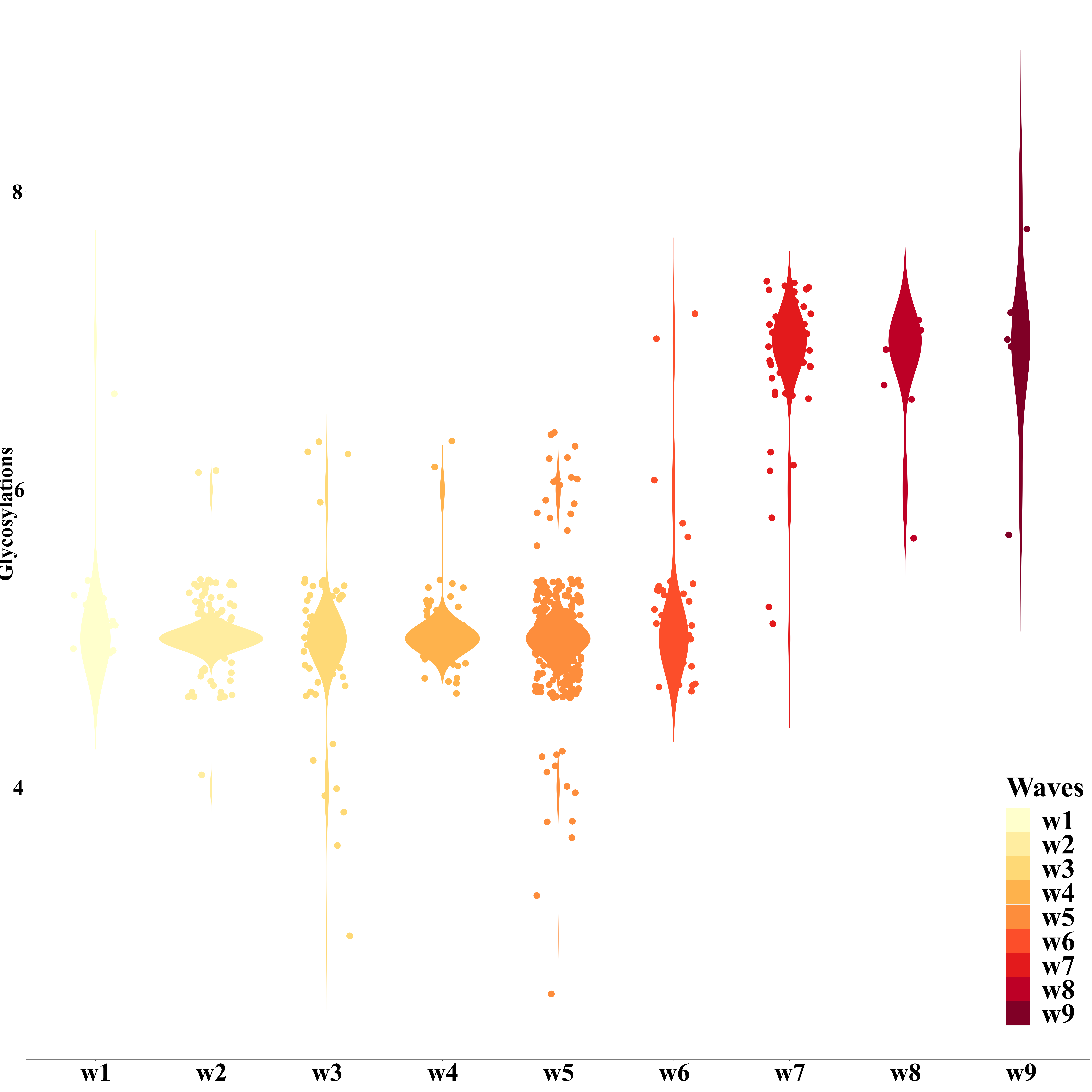

Supplement: Supplementary file 1 [file viruses-13-02524-s001.zip › Figure S4. The number of HA N-glycosylation.pdf]
